# Metabolic Mechanisms and Potential Therapies of Diabetic Cardiac Complications

**DOI:** 10.1155/2017/5130496

**Published:** 2017-01-15

**Authors:** Dake Qi, Zhengyuan Xia, Zhenwu Zhuang

**Affiliations:** ^1^Division of Biomedical Sciences, Faculty of Medicine, Memorial University of Newfoundland, 300 Prince Philip Drive, St. John's, NL, Canada A1B 3V6; ^2^Shanghai Mental Health Center, Shanghai Jiao Tong University School of Medicine, Shanghai, China; ^3^Department of Anesthesiology, The University of Hong Kong, 102 Pokfulam Road, Pokfulam, Hong Kong; ^4^Department of Anesthesiology, The Second Affiliated Hospital & Yuying Children's Hospital of Wenzhou Medical University, Wenzhou, Zhejiang 325027, China; ^5^Yale University School of Medicine, 330 Cedar Street, New Haven, CT 06520, USA

Metabolic abnormalities are important to mediate the incidence and development of cardiovascular diseases. In general, glucose metabolism in the heart accounts for almost 30% of cardiac energy production and the rest of energy (70%) to maintain cardiac function is generated from fatty acid oxidation. In diabetes or conditions that are associated with insulin resistance (such as obesity), glucose utilization is compromised in the heart, while fatty acid metabolism is upregulated, leading to abnormal lipid uptake and storage, referred to as “lipotoxicity” which contributes to the development of “cardiomyopathy.”

In addition, whole-body metabolic dysfunction during diabetes or insulin resistance is associated with hyperlipidemia and hypertension that will also contribute to vascular pathogenesis and coronary heart disease. Myocardial infarction or ischemia, caused by partial or complete occlusion of coronary arteries, is a leading cause of death in the world and often occurs in diabetic patients. Coronary artery reperfusion, or reflow, is the most effective clinical intervention to limit hypoxic injury but it simultaneously induces additional damage to the heart, referred to as “reperfusion injury.” Ischemia/reperfusion injury (IRI) and organ failure, especially IRI-induced remote and multiple organ failure, contribute significantly to postoperative mortality and morbidity, and reperfusion induced oxidative stress plays a critical role in this pathology in particular in subjects with diabetes [1, 2]. More importantly, the hearts in patients with diabetes are less tolerant to ischemic insult and less or not responsive to pre- or postconditioning cardioprotective interventions that are effective in nondiabetic subjects.

Thus, the mechanisms in mediating cardiac or whole-body metabolic alterations during diabetes or metabolic dysfunction have important clinical implications in the development of new therapies for diabetes relevant heart diseases such as cardiomyopathy and myocardial ischemia-reperfusion injury. For example, any mechanism that facilitates a rapid recovery of aerobic metabolism has the potential to limit ischemia-reperfusion injury in the heart.

In this special issue, P. C. Rezende et al. evaluated the possible influence of diabetes in myocardial ischemic preconditioning in both experimental and clinical settings of myocardial ischemia-reperfusion injury and proposed that the control of metabolic changes may restore intracellular signaling protective mechanisms in diabetes.

Atrial fibrillation (AF) is the most common abnormal human heart arrhythmia which imposes a substantial burden on population health, especially in patients with diabetes [3]. However, the underlying pathophysiological mechanisms of AF remain unclear. In this special issue, Y. Zhao et al. reported their novel findings that calcineurin, together with its upstream molecule, calpain 2, and downstream effector, NFAT-c3, plays a critical role in the development of AF in patients with vascular heart disease and diabetes.

Overall, we hope that the original and review articles presented in this special issue, representing the current advances in metabolic mechanisms and potential therapies of diabetic cardiovascular complication, with respect to their potential impact in cellular survival pathways and therapeutic strategies, will stimulate further exploration of this important area.
